# Descending mediastinitis: a review

**DOI:** 10.1590/S1516-31802006000500011

**Published:** 2006-09-07

**Authors:** Luis Marcelo Inaco Cirino, Fernando Melhem Elias, José Luiz Jesus de Almeida

**Keywords:** Mediastinitis, Infection, Mediastinum, Dental focal infection, Sepsis, Mediastinite, Infecção, Mediastino, Infecção focal dentária, Sepse

## Abstract

**CONTEXT:**

Mediastinitis is an inflammation of connective tissue that involves mediastinal structures. When the condition has an infectious origin located in the cervical or oral region, it is termed "descending mediastinitis" (DM).

**DATA SOURCES:**

The subject was examined in the light of the authors' own experiences and by reviewing the literature available on the subject. The Medline, Lilacs and Cochrane databases were searched for articles, without time limits, screening for the term "descending mediastinitis". The languages used were English and Spanish.

**DATA SYNTHESIS:**

There are three main fascial pathways by which oral or cervical infections can reach the mediastinum: pretracheal, lateropharyngeal and retropharyngeal. About 70% of DM cases occur via the retropharyngeal pathway. The mortality rate is about 50%. According to infection extent, as seen using computed tomography, DM can be classified as focal (type I) or diffuse (type II). The clinical manifestations are nonspecific and resemble other systemic infections or septic conditions. The primary treatment for DM consists of antibiotics and surgical drainage. There are several approaches to treating DM; the choice of approach depends on the DM type and the surgeon's experience. In spite of all the improvements in knowledge of the microbiology and physiopathology of the disease, controversies still exist regarding the ideal duration of antibiotic therapy and whether tracheostomy is really a necessary procedure.

**CONCLUSION:**

Since DM is a lethal condition if not promptly treated, it must always be considered to represent an emergency situation.

## RESUMO

**CONTEXTO:**

Mediastinite é um processo inflamatório do tecido conectivo que envolve as estruturas mediastinais. Quando essa condição é causada por uma infecção em sítio cérvico-oral, a inflamação mediastinal é dita mediastinite descendente (MD).

**FONTE DE DADOS:**

O assunto foi examinado através de revisão da literatura disponível e à luz da experiência dos autores. Os bancos de dados Medline, Lilacs e Cochrane foram pesquisados, sem limite de tempo, através do termo "descending mediastinitis". As línguas utilizadas foram inglês e espanhol.

**SÍNTESE DOS DADOS:**

Existem três vias fasciais principais pelas quais um foco infecioso em região cérvicooral pode se espalhar para o mediastino: pré-traqueal, latero-faríngeo e retrofaríngeo. Cerca de 70% dos casos de MD ocorrem através da via retrofaríngea. O índice de mortalidade situa-se ao redor de 50%. De acordo com a extensão da infecção e baseado nos achados de tomografia computadorizada (TC), MD pode ser classificada como focal (tipo I) e difusa (tipo II). As manifestações clínicas são inespecíficas e semelhantes às de outras infecções sistêmicas. O tratamento primário da MD consiste em antibióticos e drenagem cirúrgica. Existem diversas formas de abordagem no tratamento cirúrgico da MD; a escolha de qual via será utilizada depende do tipo de MD e da experiência do cirurgião. Apesar de todo o avanço no conhecimento da microbiologia e fisiopatologia da doença, ainda há controvérsias quanto à duração ideal da antibioticoterapia e à necessidade de se realizar traqueostomia nos pacientes portadores de MD.

**CONCLUSÃO:**

Como a MD é uma condição rapidamente fatal se não diagnosticada e tratada a tempo, ela deve ser sempre considerada uma emergência médica.

## INTRODUCTION

Mediastinitis can be defined as an inflammation of connective tissue that involves mediastinal structures and fills interpleural spaces.^1,2^ It can be secondary to infectious or non-infectious causes and, depending on the etiology, it can be acute or chronic. In its acute form, it is a life-threatening condition if not diagnosed early or if treated inadequately.^1^ The majority of cases are associated with cardiovascular operations (affecting 1 to 2% of heart surgery patients).^3,4^ However, other etiologies such as esophageal perforation, tracheobronchial perforation, mediastinal extension of pulmonary infections or mediastinal extension of head and neck infections are also possible.^5,6^ When the condition has an infectious origin located in the cervical or oral region, the mediastinal inflammation is called "descending mediastinitis" (DM). In this case, it is characterized by acute polymicrobial infection with extensive fascial necrosis that may spread toward the skin and underlying muscles.^2^ One peculiarity of this type of infection is its capacity to affect several anatomical zones, thereby provoking muscle and fascia necrosis, cellulitis, abscess formation and systemic toxicity induction.

The conditions that can lead to descending mediastinitis include, for example, retropharyngeal abscess, Ludwig's angina and odontogenic infection.^7,8^ Since descending mediastinitis is a lethal condition if not promptly treated, it must be considered to represent an emergency situation.

## DATA SOURCES

The subject was examined in the light of the authors' own experiences and by reviewing the literature available on the subject. The Medline, Lilacs and Cochrane databases were searched for articles, without time limits, screening for the term "descending mediastinitis". From this, 160 articles in all languages were found, of which 94 in English and Spanish were selected. Among these, there were 54 case reports (58%), 28 original articles (30%, including 11 case-series studies and one clinical trial), 10 letters to editors (11%) and one review (1%). No meta-analyses on descending mediastinitis were found. The articles were then read, and all the information we considered relevant for accomplishing the aim of this review was used. Articles with repeated information were rejected.

### Anatomical Considerations

During the fetal period, the neck, chest and abdomen communicate with each other and the mediastinum is the anatomical passageway. The communicating pathways between these anatomical spaces progressively close as the cervicothoracic organs form and as the body elongates cephalocaudally. Nevertheless, at adult ages, vestiges of these pathways can, under special conditions, allow orocervical infection to reach the thoracic cavity and go as far as the mediastinum.^8^

The mediastinum is bordered by the thoracic inlet superiorly, the diaphragm inferiorly, the sternum anteriorly, the vertebral column posteriorly, and the parietal pleura laterally. The three major divisions of the mediastinum are the anterior mediastinum, middle mediastinum and posterior mediastinum. The structures in the anterior mediastinum include the aortic arch and branches, the great veins, the lymphatics and the thymus gland. The middle mediastinum contains the bronchi, the heart and pericardium, the hila of both lungs, lymph nodes, phrenic nerves and the trachea. In turn, the posterior mediastinum includes the azygos vein, the descending aorta, the esophagus, lymph nodes, the thoracic duct and the vagus and sympathetic nerves.

The deep fascia of the neck is divided into three layers,^9^ which in turn divide the deep neck into three major fascial pathways by which oropharyngeal infections can spread towards the mediastinum (Table 1). The three layers are the pretracheal or superficial, visceral and prevertebral layers. In turn, there are three main fascial pathways. The first of these is the pretracheal pathway, which is anterior to the trachea and ends in the anterior mediastinum at the level of the carina. This space is limited superiorly by the thyroid cartilage and is the most superficial of these spaces. The second is the lateropharyngeal pathway, which extends from the base of the skull to the aortic arch and drains into the middle mediastinum. This is formed by fusion of the major layers of the cervical fascia, and it has communication with all the cervicofascial spaces. It is also called the "perivascular space", because it is surrounded by the carotid sheath and thus contains the carotid artery, internal jugular vein and vagus nerve. Finally, the retropharyngeal pathway is located between the esophagus and spine and is also called the "prevertebral" or "retrovisceral" space.^1,10^ This interfascial space starts at the C6 level of the spine and continues as far as the T1 level (where the alar fascia joins the inferior constrictor muscles of the pharynx); from that point onwards, the so-called "danger space" starts. This name is given because this space is patent from the skull base to the diaphragm, thereby allowing the spread of infection to the mediastinum. When infection reaches this level, the prognosis is usually poor.

**Table 1:** Main fascial pathways of the neck

Pretracheal	Lateropharyngeal	Retropharyngeal
anterior to the trachea and limited superiorly by the thyroid cartilage the most superficial ends in the anterior mediastinum at the level of the carina	also called "perivascular space" formed by fusion of the major layers of the cervical fascia extends from the base of the skull to the aortic arch drains into the middle mediastinum	also called the "prevertebral" or "retrovisceral space" located between the esophagus and spine starts at the C6 level of spine and continues as far as the T1 level

**Table 2:** Causes of descending mediastinitis^12,13^

Odontogenic infection (40-60%)
Retropharyngeal abscess (14%)
Peritonsillar abscess (11%)
Cervical lymphadenitis (7%)
Clavicular osteomyelitis (7%)
Traumatic endotracheal intubation (7%)
External trauma (5%)
Intravenous drug abuse, parotitis and thyroiditis

**Table 3:** Classification of descending mediastinitis based on radiological findings^18^

Type I (focal type)	Type II (diffuse type)	
Type IIa	Type IIb
infection is located in the superior mediastinal space above the tracheal bifurcation	infection is still located in the inferior anterior mediastinum	infectious process reaches the inferior posterior mediastinum

About 70% of the cases of DM occur through the retropharyngeal pathway^9-11^ and 8% occur via the pretracheal route.^9^ The latter is more common in infections originating from thyroid gland.^9^ The remainder of the cases occur via perivascular spreading and, in these cases, the process is frequently complicated with arterial hemorrhage. In general, pharyngeal abscesses spread into the retropharyngeal space to reach the posterior mediastinum, whereas submental and submaxillary abscesses spread towards the anterior mediastinum.^12^ It is important to remember that transdiaphragmatic spread via either the esophageal hiatus or the vena cava foramen may also occur, especially in immunocompromised patients.^2^

### Epidemiology and classification

DM mainly affects young adults. The median age is 36 years and 86% of the patients are men.^13^ Odontogenic infection is the most common cause of descending mediastinitis,^8,14^ especially when the second and third lower molars are involved. It accounts for 40-60% of the cases. The second most common cause is retropharyngeal abscess (14%). Peritonsillar abscesses makes up 11% of the etiologies. Either retropharyngeal or peritonsillar abscess may cause violation of the lateropharyngeal spaces and downward spread of the infection to the mediastinum.^12^ Less common causes include cervical lymphadenitis (7%), traumatic endotracheal intubation (7%), clavicular osteomyelitis (7%), external trauma (5%), intravenous drug abuse, parotitis and thyroiditis^13^ (Table 2). It is believed that conditions like diabetes, alcoholism, neoplasms and radionecrosis are risk factors for the development of DM.^15,16^ In addition, age greater than 70 years and diabetes have been associated with worse prognosis. Poor oral hygiene, malnutrition and long-term corticotherapy are also factors that might worsen the outcomes and hinder the treatment.^17^

Endo et al.^18^ classified descending mediastinitis as focal and diffuse types (Table 3), according to the degree of dissemination revealed by computed tomography scan (CT scan). In type I (focal type), the infection is located in the superior mediastinal space above the tracheal bifurcation. In type II (diffuse type), there are two subtypes. In subtype IIA, infection is still located in the inferior anterior mediastinum; in subtype II, the infectious process has already reached the inferior posterior mediastinum.

### Physiopathology and microbiology

Oral infections are common in populations and only rarely do they lead to serious complications. Among them, DM can be considered the most severe, with a mortality rate ranging between 25% and 50%.^1,19,20^ The main causes of poor prognosis in DM cases are the difficulty in making an early diagnosis, inadequate debridement and drainage of the cervicomediastinal spaces, the patient's clinical state (which is often poor) and the rarity of this disturbance, which hinders accumulation of experience by surgeons and general practioners.^21^

The microorganisms involved may be aerobes or anaerobes. The type most commonly isolated is beta-hemolytic oral *Streptococcus* (a consequence of the fact that most DM cases are caused by odontogenic infections).^2,8^ Other organisms commonly found include *Prevotella, Peptostreptococcus, Fusobacterium, Veillonella, Actinomyces, Bacterioides, Staphylococcus* and also alpha-hemolytic *Streptococcus*.^22^ The anaerobic germs have a high affinity for the lipidic constituents of cell membranes, thus leading to lysis of muscle cells, erythrocytes and platelet cells. Pathological states that lower tissue oxygenation (diabetes or immunodeficiency) favor the spread of infection caused by anaerobic organisms. Infection by these pathogens can involve any of the mediastinal structures, causing physiological compromise by compression, bleeding, systemic sepsis, or a combination of these.^23^

Proteasis produced by *Streptococcus* and Gram-negatives anaerobes is probably related to tissue destruction.^22^ Enzymes such as fibrinolysin and coagulase lead to ischemia and favor bacterial proliferation. In turn, hyaluronidase and collagenases distort the tissue elements and supporting structures, thereby facilitating the spread of the infection through fascial spaces. The tissue destruction is explained by multiple small-vessel thromboses that cause hypoxia and extensive edema.^23^ Helped by gravity, respiration and the negative pressure in the mediastinum, pus from the orocervical spaces rapidly reaches the mediastinal area. Sometimes, gas production also occurs, which is named "non-clostridium gaseous gangrene". Both Gram-positive *cocci* and Gram-negative *bacilli* can cause tissue lesion by gas release.^23^

Deficient vascularization and rarity of cell defenses are features of cervicomediastinal spaces.^23^ Over the course of mediastinitis, a thick layer of fibrin is formed, causing decreased mobility of the mediastinal structures. With the spread of infection, an increasing area of dead space is formed under the sternum bone.^23^

### Clinical manifestations

Since clinical features depend upon the location of the infection, descending mediastinitis presents as a wide clinical spectrum, ranging from subacute forms to devastating forms that require immediate intensive care^12^ (Table 4). Chills, high-fever, tachycardia, dyspnea and non-productive cough are the main symptoms and the most common ones associated with mediastinitis.^1,8^ When the upper mediastinum is involved, retrosternal pain that radiates upwards into the neck may be present. On the other hand, when the posterior compartment of the inferior mediastinum is affected, pain originates between the scapulae and radiates around the chest.^1^ These symptoms usually appear 24-48 hours after the stimulation process. At more advanced stages, the patient may present with sepsis and hypotension, which may require large volumes of crystalloids and vasopressor medication.^14^ Decreased urine output and signs of tissue malperfusion may also be present in septic cases. The Hamman sign (crunching sound heard with a stethoscope over the precordium during systole) is usually present, although its absence does not change the likelihood of correct diagnosis. Cervical abscess can also occur; in these cases, symptoms like dysphagia, odynophagia, dysphonia, regurgitation and cervical skin edema also appear. Cranial nerve deficits are common and are usually manifested by trismus.^9^

The diagnostic criteria for descending mediastinitis, according to Estrera et al. are: 1) evidence of oropharyngeal infection; 2) radiographic characteristics of mediastinitis; 3) intraoperative or postmortem documentation of infection; and 4) establishment of a relationship between the oropharyngeal process and mediastinitis.^24^

### Diagnosis

**Figure 1:**
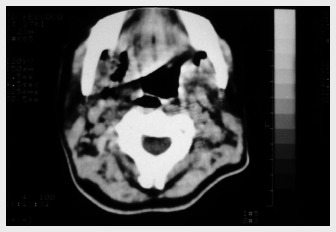
Computed tomography scan of cervical region showing bilateral lateropharyngeal and retropharyngeal collections.

**Figure 2:**
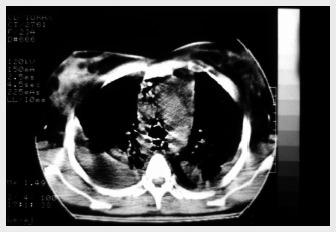
Chest computed tomography scan showing widening of mediastinal region and pleural effusion.

**Figure 3:**
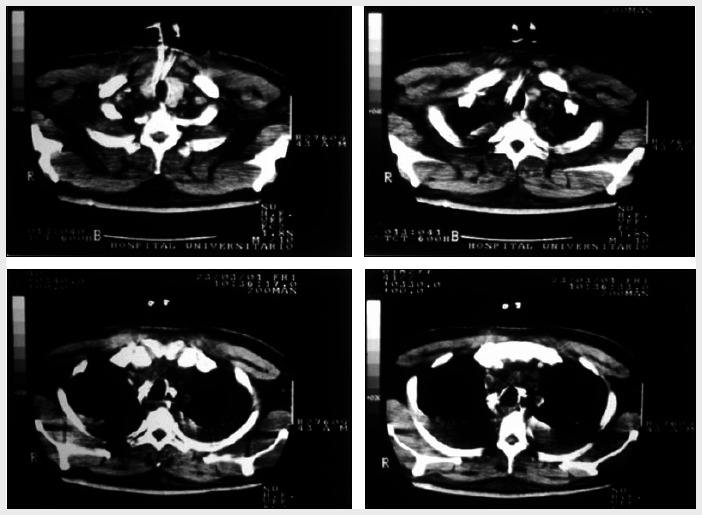
Chest computed tomography scan demonstrating mediastinal collection.

**Figure 4:**
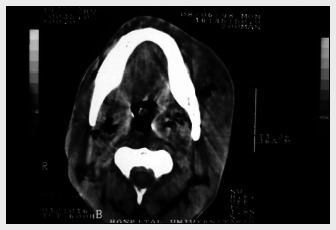
Cervical computed tomography scan showing gas in lateropharyngeal space, and also parapharyngeal and retropharyngeal bilateral abscess.

Delay in treating mediastinitis can increase morbidity and mortality. Therefore, early diagnosis is very important. Blood cells counts usually present leukocytosis with shift to the left. Hematocrit and hemoglobin may be decreased in cases of active bleeding. In patients with sepsis, the platelet count increases in the early stages and decreases in the late stages because of disseminated intravascular coagulation. The main finding from chest X-ray is pneumomediastinum with an air-fluid level, which is best viewed in lateral projection. Widening of the mediastinal shadow, pleural and pericardial effusions and lung abscess also may be found.^1,12^ However, conventional chest X-ray may mislead the diagnosing of DM. Therefore, computed tomography (CT) scan is a better and more commonly used test (Figures 1-3). Contrast-enhanced cervicothoracic CT imaging can identify DM in its early course. CT usually shows varying degrees of tissue necrosis, soft tissue infiltration, localized collections, subcutaneous emphysema and gas bands (Figure 4). CT scans make it possible to assess the spread of the infection and decide on the best surgical approach,^8,15^ and also to recognize pleural involvement and vascular complications (internal jugular vein thrombosis and carotid pseudoaneurysm). They are also useful for monitoring outcomes following surgery, and should always be repeated if there is any sign of clinical deterioration.^15^

Magnetic resonance imaging (MRI) has poor use in diagnosing mediastinitis, except in cases when the first two tests (CT scan and chest X-ray) still leave doubts regarding the patient's condition.^25^ Cultures and bacterioscopy with Gram staining are definitive diagnostic procedures. Biological material can be obtained by mediastinoscopy or subxiphoid aspiration. Several authors have pointed out that the sensitivity rate for subxiphoid aspiration ranges from 60 to 70%.^23^

### Treatment

The primary treatment for DM consists of antibiotics and surgical drainage. Correct and well-indicated antibiotic therapy is essential for effective treatment of mediastinitis. Broadspectrum antibiotics and good coverage against anaerobes should be the first choice for descending mediastinitis.^8,9,26^ Piperacillin-tazobactam and vancomycin are a good choice for empirical treatment while the culturing results are still ongoing. Another option is clindamycin plus ceftriaxone or ceftazidime. When the results are negative, empirical treatment is indicated, based on clinical signs and the existence of purulent material with fetid smell. Patients who are allergic to penicillin can receive quinolone plus clindamycin. An association of beta-lactamic plus aminoglycoside plus imidazole may be utilized after microbiological diagnosis and antibiogram (considering the great number of microorganisms that produce beta-lactamase in DM). Chemotherapy with carbapenem and metronidazole is also suggested. Hyperbaric oxygen may also be useful as adjuvant therapy.^13,23,27^

**Table 4:** Clinical manifestations of descending mediastinitis

Chills
High fever
Tachycardia
Dyspnea
Non-productive cough
Retrosternal pain
Hypotension
Hamman sign
Dysphagia
Odynophagia
Dysphonia
Regurgitation
Edema of cervical skin
Trismus

When the clinical course appears to be resistant to treatment, mycotic infection needs to be considered, as already described.^28^

However, intravenous broad-spectrum antibiotic therapy alone is not curative without surgical drainage of the cervical and mediastinal collections, with debridement and excision of all the necrotic tissue located in the affected region. Continued irrigation of the mediastinal and pleural space should be performed as well.

Four different types of surgical approach have been described (Table 5).^15,21,23,29,30^ The decision on which one to choose depends upon the stage of the infection. In the early stages, when the collection is located above the carina anteriorly, and above the fourth vertebra posteriorly (type I mediastinitis), a transcervical approach is sufficient. The incision must be wide and lateral along the sternocleidomastoid muscle.^23^ It is less invasive, but may not reach deep regions, and many procedures may be needed in order to achieve complete debridement. Nevertheless, in order to maximize the chances of obtaining a cure in more advances cases, posterolateral thoracotomy (PLT) may be performed.^29^ A focus located at the middle or inferior mediastinum, below the fourth thoracic vertebra (descending mediastinitis types IIA and IIB) should be drained by PLT, because this allows easier access to all the mediastinal structures, complete debridement and excision of necrotic tissue, and also adequate drainage of the pleural and pericardial cavities.^31^ The PLT approach is the one most utilized, although this should not be used for collections in the superior mediastinum, because of the risk of spreading the infection to the inferior spaces; this approach also allows pulmonary decortication, if necessary.^11^

**Table 5:** Surgical approaches to descending mediastinitis (DM)^21^

Transcervical	Posterolateral thoracotomy	Median sternotomy	Transthoracic
indicated for DM type I less invasive may not reach deep regions	indicated for DM types IIA and IIB allows easily access to all mediastinal structures allows pulmonary decortication	allows a good view of the operative field can also be performed in cases of bilateral collections risk of sternal osteomyelitis and dehiscence	can use either a clamshell incision or a subxiphoid approach clamshell incision yields excellent exposure of all the mediastinal structures, but is very invasive and there is a risk of damaging the phrenic nerve subxiphoid access is useful in cases of anterior mediastinal collections

Other options include median sternotomy and the transthoracic approach, using either a clamshell incision or a subxiphoid approach (also called anterior mediastinotomy). Median sternotomy allows a good view of the operative field and can also be performed in cases of bilateral collections. However, since descending dissemination occurs mainly through the prevertebral pathway, it hinders the drainage of collections by the anterior approach. Moreover, there is a high risk of sternal osteomyelitis and subsequent dehiscence of the bone.^21,29^ In addition, access to the posterobasal compartments of the chest is difficult when this approach is used. On the other hand, a subxiphoid transthoracic approach may be useful in cases of anterior mediastinal collections, in combination with the transcervical approach (although this is less frequent).^8,11^ In some cases, a combination of transcervical and subxiphoid transthoracic approaches can be also utilized.^32^

Clamshell incision (bilateral thoracotomy plus median sternotomy) yields excellent exposure of the mediastinal structures, but it is very invasive and the risk of damaging the phrenic nerve is high.^21^ Comparing patients who underwent transcervical and transthoracic drainage, Corsten et al.^33^ demonstrated statistically significant greater survival for the transthoracic group (53% versus 81%).

Furthermore, the use of video-assisted thoracoscopy has been reported by one author,^34^ but its indication is still limited to the early stages of infection or to posterior mediastinal collections. In any of these approaches, abundant irrigation and continuous drainage with straight tube drains must be performed.^11^ Through the drains, it is also possible to carry out continuous washing with antiseptic solutions. The drains must also be constantly monitored, because they are liable to become obstructed by necrotic tissue particles. The median length of time for keeping the drain in position is around three weeks.^23^ In spite of all this care, abscesses tend to form. This is why it is recommended that control CT scans should be performed regularly, following the intervention. In cases of persistent abscesses or non-drained collections, a second surgical approach is essential in order to avoid clinical deterioration. Some authors also believe that the use of tracheostomy in order to avoid airway obstruction due to pharyngeal abscess or inflammation of the tracheal wall is necessary.^9,32,35^ Nevertheless, others believe that tracheostomy can spread the infection to non-involvedareas and hinder subsequent surgical treatment.^8,35^ However, tracheostomy should be considered in cases of prolonged mechanic ventilation.

### Complications and prognosis

The main complication of descending mediastinitis is sepsis (see the signs and symptoms above). Other complications include pneumoperitoneum, pneumothorax and pleural effusions (which can lead to empyema) and pericarditis. According to Hirai et al.,^31^ the most frequent complication other than sepsis is thoracic empyema. Hemorrhage may occur following debridement procedures due to vessel erosion. The main vascular complications are thrombosis of the internal jugular veins and carotid pseudoaneurysm.^12^ Moreover, aortopulmonary fistula, aspiration pneumonia, epidural abscess and adult respiratory distress syndrome have also been reported.^10,15,32^

DM is a severe condition. The mortality rate (about 50%) is still high and few changes have been observed over the past few years. The outcome depends upon the degree of infection and the patient's underlying disease, and also on the comorbidities (such as diabetes, HIV infection etc.). The causes of death are multiple, ranging from septic shock and respiratory insufficiency to gastrointestinal hemorrhages.^12^ The most important factor for improving the clinical course, and the course that must be pursued by the healthcare team, is early detection and readily available aggressive treatment.

### Controversies and perspectives

The correct surgical approach in each case, the indication of tracheostomy in the management of the main airway, the role of therapy using hyperbaric oxygen, and the need to remove all the skin above the infected region are still controversial issues.^36^ The future for the utilization of video-assisted thoracoscopy in the surgical treatment of mediastinitis remains to be determined. Furthermore, there is a lack of studies on the duration of antibiotic therapy and the ideal time to close the wound.
